# Intercultural communication: An issue of modern times

**DOI:** 10.1192/j.eurpsy.2021.1805

**Published:** 2021-08-13

**Authors:** M. Jesus

**Affiliations:** Centro De Responsabilidade Integrada De Psiquiatria, Centro Hospitalar e Universitário de Coimbra, Coimbra, Portugal

**Keywords:** cultural, communication, Globalization

## Abstract

**Introduction:**

Globalization as lead to a more heterogeneous population than ever which makes intercultural communication an issue of modern times. Although this is positive in many ways, the differences in culture and beliefs, as well as a linguist barrier may impair clinical communication.

**Objectives:**

The authors aim to shed light in the issues regarding intercultural communication.

**Methods:**

Review of the literature including studies focusing on the the various facets of intercultural communication.

**Results:**

People from different cultural backgrounds have less access to health care and are less referenced to specialized care. Also, these patients report less satisfaction after their appointments. Although language proficiency in pointed as one of the most determinant factors, acceptance and comprehension of the patient beliefs regarding health and disease seems to play a very important role. Different cultures express symptoms differently and have different expectations when meeting a doctor. When these factors are overlooked, the doctor-patient relationship suffers and so does the treatment adherence. Doctors tend to have an identical approach to intercultural patients and native patients and to evaluate their interview as very positive, even when the same doesn’t happen with the patients.

**Conclusions:**

Although the difficulties regarding intercultural communication are widely known, most doctors fail adequate their interventions to the specific needs of their patients, not taking into consideration their different beliefs and expectations. This raises very important questions as patient dissatisfaction leads to failure to report symptoms and consequent misdiagnosis and non-compliance to the proposed treatment which ultimately results in a less efficient health care in these populations.

**Disclosure:**

No significant relationships.

